# The environmental impact of beef and ultra-processed food consumption in Brazil

**DOI:** 10.1017/S1368980023002975

**Published:** 2024-01-04

**Authors:** Gabriela Lopes da Cruz, Maria Laura da Costa Louzada, Jacqueline Tereza da Silva, Josefa Maria Fellegger Garzillo, Fernanda Rauber, Ximena Schmidt Rivera, Christian Reynolds, Renata Bertazzi Levy

**Affiliations:** 1 Department of Nutrition, School of Public Health, University of São Paulo, São Paulo, Brazil; 2 Center for Epidemiological Research in Nutrition and Health (NUPENS), University of São Paulo, São Paulo, Brazil; 3 Global Academy of Agriculture and Food Systems, The University of Edinburgh, Edinburgh, UK; 4 Department of Preventive Medicine, School of Medicine, University of São Paulo, São Paulo, Brazil; 5 Equitable Development and Resilience Research Group, Department of Chemical Engineering, College of Engineering, Design and Physical Science, Brunel University London, London, UK; 6 Centre for Food Policy, City University, London, UK; 7 Department of Geography, University of Sheffield, Sheffield, UK

**Keywords:** Ultra-processed foods, Carbon footprint, Water use, Nutritional epidemiology, Brazil

## Abstract

**Objective:**

This study evaluated the independent and combined environmental impacts of the consumption of beef and ultra-processed foods in Brazil.

**Design:**

Cross-sectional study.

**Setting:**

Brazil.

**Participants:**

We used food purchases data from a national household budget survey conducted between July 2017 and July 2018, representing all Brazilian households. Food purchases were converted into energy, carbon footprints and water footprints. Multiple linear regression models were used to assess the association between quintiles of beef and ultra-processed foods in total energy purchases and the environmental footprints, controlling for sociodemographic variables.

**Results:**

Both beef and ultra-processed foods had a significant linear association with carbon and water footprints (*P* < 0·01) in crude and adjusted models. In the crude upper quintile of beef purchases, carbon and water footprints were 47·7 % and 30·8 % higher, respectively, compared to the lower quintile. The upper quintile of ultra-processed food purchases showed carbon and water footprints 14·4 % and 22·8 % higher, respectively, than the lower quintile. The greatest reduction in environmental footprints would occur when both beef and ultra-processed food purchases are decreased, resulting in a 21·1 % reduction in carbon footprint and a 20·0 % reduction in water footprint.

**Conclusions:**

Although the environmental footprints associated with beef consumption are higher, dietary patterns with lower consumption of beef and ultra-processed foods combined showed the greatest reduction in carbon and water footprints in Brazil. The high consumption of beef and ultra-processed foods is harmful to human health, as well as to the environment; thus, their reduction is beneficial to both.

Healthy eating results from a sustainable food system that considers the impact of food production and consumption on both human and planetary health. This concept is supported by several international entities such as the FAO of the UN and the WHO^(1,2)^, as well as the EAT-Lancet Commission’s recommendations^(3)^ and the UN Sustainable Development Goals^(4)^. The Brazilian dietary guidelines state that a healthy diet must promote people’s health and well-being and protect natural resources and biodiversity^(5)^.

Radical changes in the globalised food system over the last decades^(6)^, however, seem to point in the opposite direction. Dietary factors are responsible for 50 % of mortality from non-communicable diseases^(7)^ and a third of all human-caused greenhouse gas emissions (GHG)^(8)^. It is widely recognised that these dietary changes are related to the increased consumption of ultra-processed foods and animal-source foods, associated with a low diversity of plant-based foods^(3,9,10)^.

The livestock sector is responsible for 14·5 % of the human-induced GHG emissions, with beef production accounting for 41 % of the sector’s emissions^(11)^. Beef cattle also represent the largest contribution (33 %) to the global water footprint of farm animal production^(12)^, while red meat consumption is associated with an increased risk of death from any cause and CVD and related to other chronic diseases such as cancer^(13,14)^.

Systematic reviews have shown that dietary patterns rich in ultra-processed foods are harmful to health, being associated with an increased risk of obesity and non-communicable diseases such as CHD, depression, cancer in general, and all-cause mortality^(15–17)^ and deterioration of the nutritional dietary profile^(18)^. Moreover, studies on the environmental impact of ultra-processed foods are still emerging.

Recent studies in Brazil reported parallel increases in household purchases of ultra-processed foods and total dietary GHG and water use over a period of 30 years in eleven metropolitan areas^(19)^ and a dose-response association between quintiles of ultra-processed food intake and the dietary water footprint^(20)^. However, these studies have not yet explored the effects of ultra-processed food and meat consumption on environmental indicators across the country.

Based on data from the most recent national household budget survey undertaken in Brazil, this study evaluated, for the first time, the independent and combined environmental impacts of the consumption of beef and ultra-processed foods in Brazil, quantified through carbon and water footprints.

## Methods

### Data source

All data analysed in this study come from the Household Budget Survey (Pesquisa de Orçamentos Familiares) conducted by the Brazilian Institute of Geography and Statistics (Instituto Brasileiro de Geografia e Estatística) from July 2017 to July 2018^(21)^. The total sample of households was 57 920. The survey used a complex two-stage cluster sampling plan, involving the random selection of census tracts in the first stage and households in the second. The census tracts come from the Instituto Brasileiro de Geografia e Estatística’s master sample, grouped into strata of households with high geographical and socioeconomic homogeneity. For the construction of the strata, the following were considered: the geographic location of the sector; the situation of the household (urban or rural for samples with national representation); and, within each geographic locus, the spectrum of socioeconomic variation through the income of the individual responsible for the household.

The estimates obtained in the survey represent the following domains: the country, the five large regions (North, Northeast, Southeast, South, and Midwest), the situation (urban or rural), the twenty-six federal units and the Distrito Federal, the nine metropolitan regions, and the twenty-six state capitals.

For the present study, the household clusters generated in the sampling plan (strata) were used as the unit of analysis. The 57 920 households resulted in 575 strata with an average of 86·5 households per stratum (ranging from 16 to 524).

The data analysed in this study refer to the purchase of food items for consumption by households over seven consecutive days. Members of the household recorded all the purchased items, assisted by the Instituto Brasileiro de Geografia e Estatística interviewer if necessary. These items did not include foods and beverages consumed outside the house. Information on the total expenses with food out-of-home was collected.

The data collection was carried out over 12 months guaranteeing representativeness for all four year’s seasons. Details of the Pesquisa de Orçamentos Familiares sampling process can be found in Instituto Brasileiro de Geografia e Estatística’s publication [https://biblioteca.ibge.gov.br/visualizacao/livros/liv101670.pdf].

### Assessment of food purchase

The total amount of food and beverages acquired by strata was divided by seven to obtain the daily acquisition and then divided by the total number of residents in the strata to obtain the per capita estimate. To estimate the energy (calories) purchased from each food item, the amount of inedible parts of foods (such as seeds, husks, bones, etc.) was excluded using correction factors^(22)^. The amounts of edible parts were converted from kilograms or litres into calories using the Brazilian Food Composition Table of the University of São Paulo, Food Research Center (FoRC), Version 7.0. São Paulo, 2019 [Access available at: http://www.fcf.usp.br/tbca].

We categorised all the foods and beverages according to the Nova classification system into (i) unprocessed or minimally processed foods, (ii) processed culinary ingredients, (iii) processed foods and (iv) ultra-processed foods, and respective subgroups^(23)^. The Nova classification system groups foods according to the extent and purpose of industrial food processing. The first group comprises unprocessed or minimally processed foods, which are edible parts of plants or animals, mushrooms, and algae obtained from nature or subjected to the removal of inedible or unwanted parts, dehydration, milling, fractionation, roasting, pasteurisation, freezing and other processes that do not involve the addition of substances. The second group is composed of processed culinary ingredients, comprising substances extracted directly from foods of the first group (unprocessed or minimally processed foods) or nature, such as sugar, salt, oils and fats. The third group consists of processed foods, industrially made items obtained by adding ingredients from the second food group (processed culinary ingredients) to the first food group (unprocessed or minimally processed foods), creating new foods such as bread and cheese. The fourth group, ultra-processed foods, is made of highly industrialised formulations often rich in sugar, salt and fat, containing little or no unprocessed or minimally processed foods in its whole form, and characterised by the presence of food additives such as colours, flavours, flavour enhancers, emulsifiers, thickeners and other cosmetic chemical substances. Examples are sweetened beverages, ready-to-eat meals and cookies.

The exposure variables used in the study were the percentage of total energy intake from all ultra-processed foods (regardless of their animal or vegetal source) and the percentage of total energy intake from all beef food items (regardless of their Nova group). The latter includes fresh beef cuts and offal – subgroup beef, group (i); dried beef, jerky and sun-dried beef – subgroup salted, cured, smoked beef – group (iii); and hamburgers, pâté and other products mainly produced from reconstituted beef – subgroup reconstituted beef products, group (iv).

### Assessment of the environmental impact

This study considers two environmental impact indicators, namely carbon footprint and water footprint. Carbon footprint refers to the quantification of the greenhouse gases – carbon dioxide, methane, nitrous oxide, hydrofluorocarbons, perfluorocarbons, sulphur hexafluoride among others – directly or indirectly caused by any activity related to the life cycle of a (food) product^(24)^. This indicator is expressed in mass, in this case grams, of carbon dioxide equivalent (gCO_2_eq). Water footprint measures the total amount of freshwater directly or indirectly used during the life cycle of food, defined by the sum of surface water (blue water), rainwater (green water) and water needed to assimilate the load of pollutants associated with production and consumption systems (grey water). The water footprint is expressed in litres^(25)^.

The environmental impacts of purchased foods were estimated using the Brazilian food database as background data, which accounts for the carbon and water footprint of Brazilian food items per mass or volume^(26)^. This database has a cradle-to-retail scope, including the complete life cycle of a food product from the farm to the point of sale [Access available at: ·https://doi.org/10.17605/OSF.IO/GS4CY].

The environmental indicator of each purchased food is estimated by multiplying the environmental impact of each food per mass or volume (e.g. g CO_2_eq/kg_food_ – *L*_food_, *L*//kg_food_ – *L*_food_) by the total weight (e.g. kg_food_) or volume (*L*_food_) of each purchased food, which englobes the discarded and/or inedible parts (such as husks, seeds, bones) since they are inherent to the production of the edible fraction; thus, their impact is inseparable. Environmental impacts of cooking were not considered.

### Data analysis

First, we calculated the 2017–2018 Brazilian total dietary energy household food availability (kcal/person-day) and the corresponding carbon and water footprints (gCO_2_eq and l/person-day). Then, we calculated the percentage contribution of each Nova food group and subgroup to the total energy availability and to the total carbon and water footprints. For each food group and subgroup, a ratio was calculated between the mean percentage of carbon or water footprint and the mean percentage of dietary energy availability (% carbon footprint/% dietary energy availability, % water footprint/% dietary energy availability). Ratios equal to one identify food groups and subgroups with footprints per energy consumed identical to the food availability. Ratios above one and below one identify food groups and subgroups with footprints, per unit of energy, higher and lower than the diet, respectively.

Next, we calculated the mean carbon and water footprints per 1000 kcal of total daily food purchases and assessed their variation according to quintiles of the beef and ultra-processed foods contributions to daily energy intake. Crude and adjusted (for income, area of residence [urban and rural], macro-region of the country and percentage of out-of-home expenses on total household expenditure) linear regressions were used to calculate mean footprints and test their association with quintiles of beef and ultra-processed food contributions to caloric intake.

Finally, we calculated the predicted Brazilian daily mean carbon and water footprints per 1000 kcal if all food purchases were identical to those found in the first quintile of ultra-processed foods contribution (first scenario), identical to those found in the first quintile of beef contribution (second scenario) or identical to those found in the first quintile of ultra-processed foods and in the first quintile of beef contribution (third scenario). Values were predicted using multiple linear regression analysis adjusted for income, area, region and out-of-home expenses. We used the Wald test to assess multiplicative interactions between the quintiles of beef and ultra-processed foods purchases to the final footprint value. A *P*-value lower than 0·05 was considered significant.

Weighting factors were used according to sample structure, which allows the extrapolation of results to the Brazilian population. All analyses were performed using the Stata statistical package version 15.1.

## Results

Table 1 shows the distribution of the total household food energy availability (1221·5 kcal/person-day) and the distribution of the corresponding carbon footprint (2139·3 gCO_2_eq/person-day) and water footprint (1963·8 l/person-day) according to the Nova food groups and subgroups.

**Table 1.** Energy content and environmental footprints of food purchases according to Nova food groups and subgroups, Brazilian households, 2017–2018*,†

Nova food groups and subgroups	Energy				Carbon footprint					Water footprint				
	Kcal/person-day‡		% of total (a)		gCO2eq/person-day		% of total (b)		Ratio (b)/(a)	l/person-day		% of total (c)		Ratio (c)/(a)
	Mean	se	Mean	se	Mean	se	Mean	se		Mean	se	Mean	se	
Unprocessed or minimally processed foods	597·1	10·1	48·7	0·4	1588·8	27·4	73·9	0·5	1·5	1316·2	21·3	66·9	0·5	1·4
Rice and other cereals	198·9	5·3	16·2	0·3	147·92	3·9	7·2	0·2	0·4	79·7	2	4·2	0·1	0·3
Beef	57·7	1·4	4·8	0·1	955·38	21·3	43·7	0·5	9·1	636·2	14·4	31·9	0·4	6·7
Milk	57·5	1·1	4·8	0·1	127·12	2·5	6·1	0·1	1·3	111·6	2·6	5·7	0·1	1·2
Cassava, wheat and corn flour	55·7	2·8	4·3	0·2	8·58	0·5	0·4	0	0·1	25·4	1·3	1·3	0·1	0·3
Beans	50·6	1·3	4·1	0·1	4·64	0·1	0·2	0	0·1	58·6	1·5	3·1	0·1	0·7
Poultry	49·7	1·4	4·1	0·1	119·09	2·9	5·7	0·1	1·4	158·8	3·9	8·2	0·2	2·0
Fruits	34·1	0·9	2·8	0·1	54·01	1·7	2·5	0·1	0·9	58·1	1·7	2·9	0·1	1·0
Pasta	29·4	0·7	2·4	0·1	15·94	0·5	0·8	0	0·3	14·4	0·4	0·8	0	0·3
Vegetables, roots, and tubers	26·8	0·6	2·2	0	20·91	0·5	1·0	0	0·4	37·7	0·9	1·9	0	0·9
Pork	12·1	0·5	1·0	0	34·15	1·6	1·6	0·1	1·7	55·0	2·2	2·8	0·1	2·9
Eggs	11·3	0·3	0·9	0	31·00	0·8	1·5	0	1·6	28·3	0·7	1·5	0	1·6
Fish	5·4	0·4	0·4	0	23·63	1·6	1·1	0·1	2·6	0·2	0·2	0·0	0	0·0
Other§	8·0	0·3	0·7	0	46·40	2·6	2·1	0·1	3·2	52·3	2·1	2·6	0·1	3·9
Processed culinary ingredients	268·6	6·1	21·6	0·3	47·24	1·1	2·2	0	0·1	100·8	2·3	5·2	0·1	0·2
Plant oils	133·2	3·8	10·8	0·2	26·25	0·7	1·3	0	0·1	70·2	1·9	3·6	0·1	0·3
Sugar	117·4	3·1	9·4	0·2	12·63	0·3	0·6	0	0·1	20·2	0·5	1·1	0	0·1
Animal fats	8·9	0·7	0·7	0	6·63	0·6	0·3	0	0·4	5·3	0·4	0·3	0	0·4
Starch	8·1	0·4	0·7	0	1·37	0·1	0·1	0	0·1	4·7	0·2	0·2	0	0·4
Other¶	1·0	0·1	0·1	0	0·35	0	0·0	0	0·3	0·5	0·1	0·0	0	0·3
Processed foods	122·6	2·4	10·4	0·2	153·25	4·8	7·3	0·2	0·7	152·4	4·4	8·0	0·2	0·8
Bread	81·3	1·9	7·0	0·2	15·83	0·4	0·8	0	0·1	50·1	1·2	2·7	0·1	0·4
Cheese	17·3	0·7	1·4	0·1	46·68	1·9	2·2	0·1	1·5	31·0	1·2	1·6	0·1	1·1
Beer and wine	8·3	0·5	0·7	0	24·49	1·3	1·1	0·1	1·6	8·3	0·4	0·4	0	0·6
Salted, cured, smoked beef	3·5	0·3	0·3	0	44·54	4	2·2	0·2	7·5	41·7	3·7	2·2	0·2	7·7
Salted, cured, smoked meat other than beef	5·2	0·3	0·4	0	9·14	0·5	0·4	0	1·0	6·8	0·4	0·4	0	0·8
Other**	7·0	0·3	0·6	0	12·57	0·4	0·6	0	1·0	14·6	0·5	0·7	0	1·3
Ultra-processed foods	233·2	5	19·4	0·4	350·02	9·4	16·6	0·4	0·9	394·5	10·3	20·0	0·4	1·0
Cookies, cakes, and pies	43·3	1	3·6	0·1	21·55	0·5	1·0	0	0·3	23·9	0·5	1·2	0	0·3
Reconstituted meat products other than beef	31·6	0·8	2·6	0·1	160·61	4·3	7·6	0·2	2·9	167·8	4·2	8·6	0·2	3·3
Chocolate, ice cream, and other candy	30·3	1·1	2·5	0·1	21·16	0·7	1·0	0	0·4	94·6	3·7	4·8	0·2	1·9
Crackers	22·8	0·6	1·9	0	8·02	0·2	0·4	0	0·2	7·8	0·2	0·4	0	0·2
Margarine	21·7	0·5	1·8	0	5·32	0·1	0·3	0	0·1	4·5	0·1	0·2	0	0·1
Ready-to-eat meal	21·7	1	1·8	0·1	39·48	2·9	1·8	0·1	1·0	23·7	1·3	1·2	0·1	0·7
Sweetened beverages	21·2	0·7	1·8	0·1	27·20	1	1·3	0	0·7	24·1	0·9	1·2	0	0·7
Bread	16·8	0·6	1·4	0·1	9·15	0·3	0·4	0	0·3	7·1	0·3	0·4	0	0·3
Milk-based products	8·8	0·3	0·7	0	15·19	0·6	0·7	0	1·0	9·2	0·4	0·5	0	0·6
Sauces	8·6	0·3	0·7	0	25·39	1	1·2	0	1·6	18·4	0·7	0·9	0	1·3
Reconstituted beef products	1·4	0·1	0·1	0	8·77	0·7	0·4	0	3·5	6·9	0·5	0·4	0	3·0
Other††	4·9	0·2	0·4	0	8·18	0·4	0·4	0	1·0	6·3	0·2	0·3	0	0·8
Total	1221·5	15·9	100·0		2139·3	32·3	100·0			1963·8	27·4	100·0		

* Dietary data source: Brazilian Food Composition Table.

† Footprint data source: Table of carbon, water and ecological footprints for each 100g of foods and culinary preparations consumed in Brazil^(26)^.

‡ Individual daily mean of purchased food items for consumption by households, excluding those food items consumed outside the home.

§ Coffee, tea, nuts, seafood, exotic meat and freshly prepared dishes.

¶ Salt, honey, syrup, vinegar.

** Canned/tinned fruits and vegetables and salted fish, salted nuts.

†† Distilled alcoholic beverages, condiments and breakfast cereal.

Unprocessed or minimally processed foods contributed to 48·7 % of total food energy availability, 73·9 % of total carbon footprint and 66·9 % of total water footprint, i.e. with ratios footprint/energy of 1·5 and 1·4 ratio, respectively. Processed culinary ingredients contributed to 21·6 % of total energy availability and only 2·2 % of total carbon footprint (0·1 footprint/energy ratio) and 5·2 % of total water footprint (0·2 footprint/energy ratio). Processed foods contributed to 10·4 % of total energy availability, 7·3 % of total carbon footprint (0·7 footprint/energy ratio), and 8·0 % of total water footprint (0·8 footprint/energy ratio). Finally, ultra-processed foods contributed to 19·4 % of total energy availability, 16·6 % of total carbon footprint and 20·0 % of total water footprint with ratios footprint/energy of 0·9 and 1·0, respectively.

Among unprocessed or minimally processed foods, the food subgroup with the higher footprint/energy ratio was beef (9·1 and 6·7 for carbon and water footprints, respectively); among processed foods was salted, cured, smoked beef (ratios of 7·5 and 7·7, respectively), and among ultra-processed foods was reconstituted beef products (ratios of 3·5 and 3·0, respectively).

Total beef food purchases (including beef in unprocessed or minimally processed, processed, and ultra-processed food groups) contributed to 5·2 % of total household food energy availability and to 46·3 % of total carbon footprint and to 34·5 % of total water footprint (ratios energy/footprint of 8·9 for carbon and 6·6 for water footprint).

Table 2 shows the association between quintiles of the energy contributions of beef and ultra-processed foods and the footprints of total food purchases per 1000 kcal. The contribution of both beef and ultra-processed foods showed a significant linear association with carbon and water footprints (*P* for trend <0·01) in crude and adjusted models. The carbon and water footprints observed in the crude upper ultra-processed food quintile were 14·4 % and 22·8 % higher than in the lower quintile. Carbon and water footprints in the crude beef upper quintile exceeded by 47·7 % and 30·8 %, respectively, the same footprints seen in the lower quintile.

**Table 2.** Multiple adjusted environmental footprints of food purchases by quintiles of the contribution of ultra-processed foods and beef. Brazilian households, 2017–2018 **†**

Environmental footprints	Quintiles of the contribution of beef to total energy **§**										Quintiles of the contribution of ultra-processed foods to total energy **¶**									
	1		2		3		4		5		1		2		3		4		5	
	Mean	se	Mean	se	Mean	se	Mean	se	Mean	se	Mean	se	Mean	se	Mean	se	Mean	se	Mean	se
Carbon footprint (gCO_2_eq/1000 kcal)																				
Crude	1462·5	41·4	1622·0	23·2	1774·7	35·3	1836·1	22·4	2160·2	38·5 *	1702·3	37·6	1671·1	32·9	1738·8	28·1	1793·6	44·2	1947·0	39·0 *
Adjusted‡	1449·5	28·9	1602·8	17·7	1751·2	23·2	1853·6	18·0	2198·7	38·9 *	1738	51·4	1645·7	29·2	1734·4	26·4	1798·8	42·0	1936·0	58·1 **
Water footprint (litres/1000 kcal)																				
Crude	1422·6	37·5	1534·1	22·1	1643·6	30·3	1654·6	20·6	1860·8	29·5 *	1479·0	26·3	1525·2	21·7	1619·8	20·9	1676·1	30·2	1815·6	27·5 *
Adjusted‡	1401·4	25·1	1511·1	15·9	1617·7	20·5	1674·7	14·4	1911·2	26·4 *	1531·4	37·7	1523·6	21·2	1611·8	19·0	1662·1	28·1	1785·8	42·5 *

* Linear regression *P* trend value <0·001.

** Linear regression *P* trend value <0·01.

† Footprint data source: Table of carbon, water and ecological footprints for each 100 g of foods and culinary preparations consumed in Brazil^(26)^.

‡ Adjusted for income, area, region and out-of-home expenses.

§ Percentual of beef consumption in each quintile (se): Q1 = 3·0 % (0·1); Q2 = 4·1 % (0·0 %); Q3 = 4·9 % (0·0 %); Q4 = 5·8 % (0·1 %); Q5 = 8·2 % (0·2 %).

¶ Percentual of ultra-processed foods in each quintile (se): Q1 = 10·1 % (0.2); Q2 = 15·7 % (0·2 %); Q3 = 19·9 % (0·2 %); Q4 = 23·3 % (0·2 %); Q5 = 28·1 % (0·4 %).

**Fig. 1:**
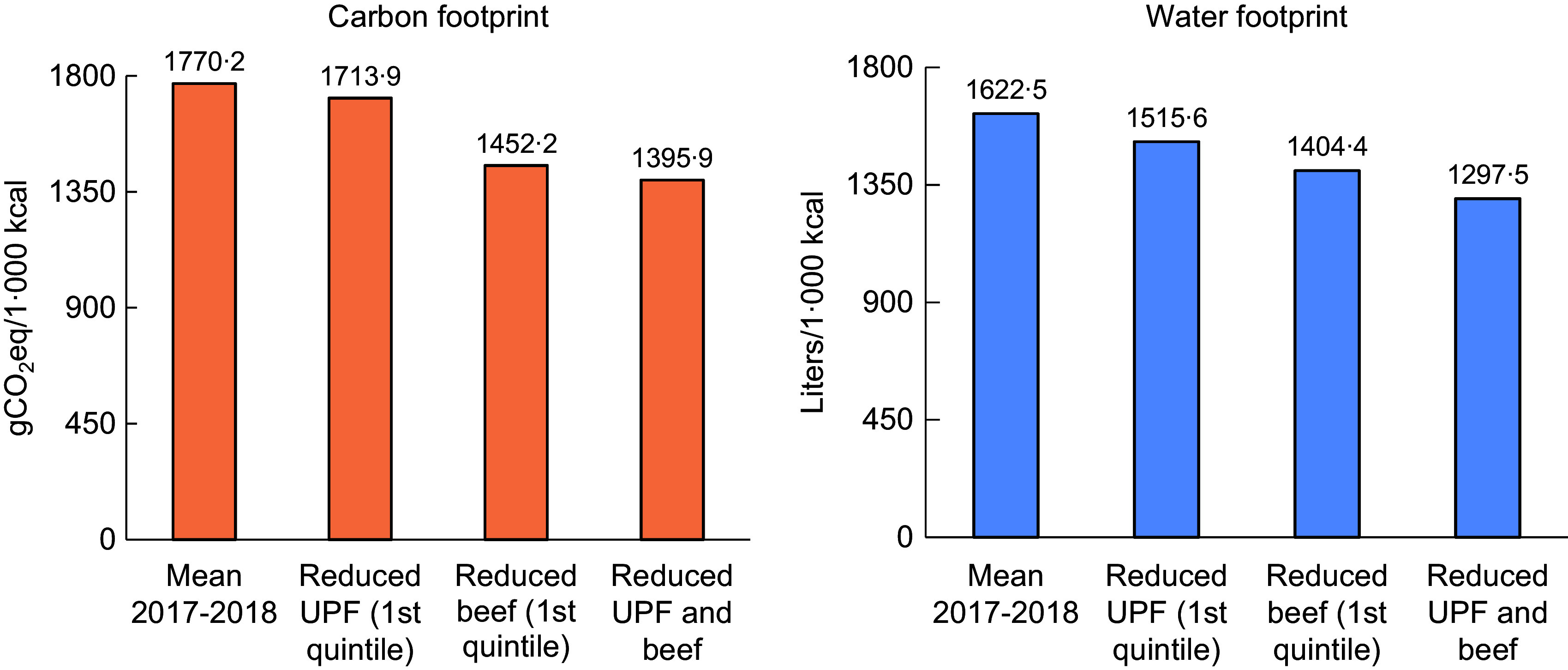
Average carbon and water footprints of food purchases in Brazil in 2017–2018 and in three different simulated scenarios according to beef and ultra-processed foods consumption. Brazilian households, 2017–2018. *Notes*: Footprint data source: Table of carbon, water and ecological footprints for each 100 g of foods and culinary preparations consumed in Brazil^(26)^. Predicted values adjusted for income, area, region and out-of-home expenses. Percentual of ultra-processed foods in the 1st quintile (s e) = 10·07 % (0·21). Percentual of beef consumption in the 1st quintile (se) = 2·99 % (0·07)

Figure 1 presents the mean carbon and water footprints of total actual food purchases by Brazilian households and the same values in three different scenarios corresponding to reduced energy contribution of ultra-processed foods (lower quintile), reduced contribution of beef (lower quintile) and reduced contribution of ultra-processed foods and beef (lower fraction).

The actual food purchases correspond to 1770·2 gCO_2_eq and 1622·5 l of water per 1000 kcal/person-day. With a reduced contribution of ultra-processed foods, the carbon and water footprints would be reduced to 1713·9 gCO_2_eq (–3·2 %) and 1515·6 litres (–6·6 %) per 1000 kcal/person-day, respectively. With a reduced contribution of beef, the two footprints would reduce to 1452·2 gCO_2_eq (–18·0 %) and 1404·4 l (–13·4 %) per 1000 kcal/person-day. The highest reduction in the carbon and water footprints would be seen with the reduction in purchases of both ultra-processed foods and beef: 1395·9 gCO_2_eq and 1297·5 l per 1000 kcal/person-day, or 21·1 % and 20·0 %, respectively. The Wald test showed no significant interaction between reduced ultra-processed foods and beef contributions.

## Discussion

The present study unprecedentedly showed that the energy contribution of both beef and ultra-processed foods is directly associated with greater carbon and water footprints. Simulated scenarios showed that a reduction in beef and ultra-processed foods consumption, combined, would represent a 20 % reduction in the environmental footprints of the Brazilian diet, being the most efficient scenario for reducing environmental impacts compared to scenarios reducing only one of the food groups.

Despite representing only 5·2 % of the available calories, beef is responsible for almost half of the carbon footprint and just over a third of the water footprint of food purchases in Brazil. Furthermore, GHG and water increased as the share of beef in the daily diet got higher. These findings support the solid body of evidence indicating meat as one of the main drivers of the climate change associated with food production and consumption around the world^(27)^.

Agricultural beef production relates to a system of land degradation, deforestation, loss of biodiversity and high water consumption^(28)^. According to FAO, 14·5 % of greenhouse gases emitted by human activities are related to the livestock sector, and beef production accounts for 41 % of that^(11)^. In Brazil, livestock production and land use account for 73 % of the country’s total GHG ^(29)^. Meat production does not only affect the environment, but its excessive consumption seems to be harmful to human health. WHO classifies processed red meat (which includes ultra-processed foods) and unprocessed meat as carcinogenic and probably carcinogenic to humans, respectively^(14)^. As a cancer prevention measure, it is recommended to limit red meat consumption and avoid processed meats^(30)^. The Eat-Lancet Commission recommends for a diet that promotes human and environmental health a maximum of 30 kcal/day of red meat (14 g/d)^(3)^.

It was novel to compare the environmental impact of beef with that of ultra-processed foods. Although the impact of ultra-processed foods is considerably lower than the observed for beef, a positive association between ultra-processed foods and environmental footprints was observed, indicating a greater environmental damage in food patterns rich in ultra-processed foods.

Environmental impacts related to ultra-processed foods are complex, influenced by commercial, biological and social factors^(31)^. Many ultra-processed foods are composed of high amounts of sugar, vegetable fats and/or refined transgenic grains (also used as the basis for cattle feed) which are directly related to the three largest monocultures produced in Brazil: soy, sugar cane and corn^(32)^. Monocultures are often linked to production systems with extensive land use, pesticides and chemical fertilisers, contamination of water sources and degradation of soil quality. These conditions pose significant risks to the environment, apart from being inherent to latifundium and income concentration, a source of social problems^(33–35)^. In addition, numerous ultra-processed foods are manufactured using ingredients extracted from a handful of high-yielding plant species, contributing to agrobiodiversity loss^(36)^. Untreated food industry waste poses disposal and pollution challenges while representing a loss of valuable biomass and nutrients in the absence of proper recovery methods and technologies^(37)^.

In a study conducted in the Netherlands, it was revealed that ultra-processed foods participation on the diet was associated with higher GHG^(38)^. In Australia, discretionary foods account for a significant 35 % water use, 39 % energy use, 33 % carbon dioxide equivalent and 35 % land use of the overall diet-related life cycle^(39)^. In México, the consumption of certain groups of ultra-processed foods (fast food, sugary drinks, sugars and desserts) and processed meats contribute to a high water footprint^(40)^; a French study also found an association between the percentage of ultra-processed foods in the diet and water use^(41)^.

It is important to mention that the association between the share of ultra-processed foods in the diet and the carbon footprint found in this study was not observed in another study using dietary intake data of the Brazilian population^(20)^. This may occur due to differences between individual dietary intake and household food purchase data implications to the caloric and footprint estimation. Although it can better estimate food consumption from individual dietary data, the dietary intake data do not account for discarded food mandatory to some final preparations, particularly the processed culinary ingredients, such as cooking oil used in deep frying recipes – which is contemplated in purchases data. The budget survey also accounts for the environmental impact of wasted food that was purchased but not consumed. Differences also occur as there is a lack of detailed data on out-of-home food expenses in the household survey, although we perform analyses adjusted for the percentage of out-of-home spending due to this absence and present results per 1·000 kcal. A study conducted in urban areas of Brazil found that out-of-home food consumption was positively associated with increased atmospheric GHG, regardless of age and income^(42)^.

Besides these findings, more studies incorporating different indicators are necessary for a global picture of the impact of ultra-processed foods on the environment. Characteristics such as low price, convenience and marketing appeal can lead to a partial or complete replacement of local and traditional foods by ultra-processed ones, particularly in emerging countries, compromising the demand and insertion of small-scale farmers in the labour market, leading to other environmental and social problems^(34)^.

The consumption of ultra-processed foods has increased significantly in several countries due to recent changes in global food systems^(9)^. Brazil is following this trend with the relative consumption of available calories from ultra-processed foods rising from 14·3 % to 19·4 % between 2002 and 2018^(43)^. In that same period (2002–2018), the household purchase of beef increased by almost 40 %^(43)^, placing Brazil among the countries with the highest beef per capita availability in the world^(44)^.

The ultra-processed food industry and the beef sector are both dominant players in the global food system. Consequently, their high consumption cannot be attributed to coincidences, but can be seen as part of wider structural conditions resulting from past and current food systems^(45–47)^. An underlying factor of this scenario is the political systems that favour the rise of ultra-processed food transnationals and agribusiness^(9)^. Ultra-processed foods are produced globally by large transnational corporations wielding authority over cultivation, production, marketing and sales in food systems^(48)^. Agribusiness is one of the most powerful sectors of the Brazilian economy, with 45 % of countrywide rural lands occupied by pastures^(32)^.

Due to these facts, it is fundamental to reduce the influence of large commercial interests in the public policy to implement policies to benefit health and environment^(10)^. The paths to reduce the environmental footprints associated with beef and ultra-processed foods involve, firstly, reducing the consumption, prioritising a diet rich in unprocessed and minimally processed foods mostly plant-based, as recommended in the Dietary Guidelines for the Brazilian Population^(5)^. In Brazil, rice and beans are the most commonly consumed foods countrywide, but the consumption of both is decreasing^(43)^. As part of the traditional Brazilian food culture, and exhibiting low carbon and water footprints, as demonstrated by the findings of this study, the encouragement of its consumption is highly advantageous. Additionally, food governance measures should encourage sustainable production systems that promote a greater and accessible diversity of plant-based unprocessed and minimally processed foods^(5)^.

Secondly, to reduce the intensity of ecological use, regulations and taxes could be implemented to limit emissions and environmental damage, along with improvements in production efficiency. According to FAO, scaling up efficient practices in raising and feeding cattle can reduce up to a third of the sector’s global emissions^(11)^. Mitigation policies are encouraged by international organisations; however, the incentives provided are still fragile. Finally, actions to promote healthy and sustainable diets involve focusing on common systemic drivers of health and environment that need common actions, as proposed by the Global Syndemic report^(10)^.

As a limitation, the Household Budget Survey considers only food purchased for consumption at home, which represented about 70 % of the total food consumption in Brazil in 2017–2018^(21)^. Despite this, Louzada *et al*. showed that household food acquisition data are a proxy for food consumption in Brazil, especially for analysis focused on relative food contribution rather than absolute quantities^(49)^. Another limitation is the environmental indicators, used as the best possible environmental estimative available rather than exact measurements. Besides, a part of the environmental footprint values was calculated for international production, with a smaller fraction reflecting the food production in Brazil, due to a lack of data. In addition, the environmental footprints were estimated for foods as they are purchased, and cooking effects have not been considered in this analysis. If the effects of cooking were considered, the carbon footprint would probably be higher, as cooking can contribute as much as 61 % of GHG for individual food^(50)^.

In this paper, we studied the environmental impacts of beef consumption combined with ultra-processed foods in a novel way. The analysis of environmental impact according to the Nova classification system is a strength of the study, as characteristics of industrial food processing deepen the discussion in an internationally recognised model. It is also a strength of this study the use of a representative sample of the Brazilian population, encompassing geographic and socioeconomic differences of the country.

Healthy eating is a synergistic and intertwined concept between human and environmental health. Although the environmental footprints associated with beef consumption are higher, dietary patterns with lower consumption of beef and ultra-processed foods combined showed the greatest reduction in carbon and water footprints in Brazil. The high consumption of ultra-processed foods and beef is harmful to human health, as well as to the environment; thus, their reduction is beneficial to both.
